# Supplementary material for: Monitoring Universal Health Coverage reforms in primary health care facilities: Creating a framework, selecting and field-testing indicators in Kerala, India
Source: PLoS One. 2020 Aug 3;15(8):e0236169. doi: 10.1371/journal.pone.0236169 (PMC7398520; doi:10.1371/journal.pone.0236169)
Supplement: S1 File — (PDF) [file pone.0236169.s001.pdf]

The George Institute for Global Health India

Supplementary document (S1)

The purpose of this document is to present a shortlist of indicators with all relevant information for experts to assess their suitability for monitoring and evaluation of Kerala's Family Health Centre programme through a ranking exercise, also known as a Delphi.

What we are doing

This activity is the second round of ranking of indicators for their relevance in Kerala from the currently recommended battery of global UHC related indicators, the existing work on Aardram Monitoring and Evaluation, as well as the Government Order for Aardram.

What we need your help with

In order to arrive at a final list of indicators, we need your input, from a technical perspective ranking how appropriate these indicators are and how well they will be able to determine whether populations are being left out. Once we have received responses from you, we will collate and summarise the findings and carry out additional rounds of ranking with additional decision-makers. Once ranking is complete, we shall share the final list and information about our process with you. You should receive this in the coming months.

How to provide your input

We have this document and an excel sheet. The ranking exercise is to be done in **this document**, and information to help you is given in the excel sheet.

In this document

You will see a list of indicators on page 4 of this document. Please rate each of the indicators in terms of

a) its value in assessing health equity and providing information to policy makers about important health reforms in Kerala and

b) degree of priority that should be placed on measuring this indicator immediately. Given Kerala's current scenario, we may feel an indicator is important but cannot or should not be assessed in the coming year.

Please note that **a rank of 1 indicates the highest possible ranking of priority** (i.e. the indicator is important and/or should be monitored immediately). A **rank of 5 indicates that this is the lowest priority indicator** (i.e. the indicator is not as important and/or can be prioritised for monitoring later).

Please note that each indicator has to be ranked individually – i.e. multiple indicators can have a rank of 1 (meaning high priority). If you have ranked one indicator 1, this does not mean that the next indicator cannot also be ranked 1. Considerations and criteria for ranking are also indicated in this document.

You also have the option of adding indicators under each domain area – in which case providing a justification is required, including what the data source for the indicator could be.In case of addition of a new indicator please rank that too

In the Excel sheet

This file has three sheets.

The first sheet is LEGEND which explains all the column headings in the subsequent sheet. In the sheet INDICATOR_SHORTLIST, is the list of initial indicators currently, divided into sub-domains recommended for Universal Health Coverage under the Sustainable Development Goals indicator process as well as the Joint Learning Network on Primary Health Care. These sub-domains are:

reproductive, maternal and child health; communicable diseases; non-communicable diseases; service capacity and access; and financial risk protection.

There is also a sheet called INDICATOR_LONGLIST, which contains the entire battery of over 800 indicators, from various sources, that we have compiled for this exercise. You can identify additional indicators from here to recommend/add to the shortlist as part of your ranking. We have tried to organise these so you can sort through them looking at the FHC packages, various sources, domains and sub-domains.

How were the shortlisted indicators chosen?

The list of indicators that you see were chosen by us and through an initial mapping exercise of indicators used globally for Universal Health Coverage and reform of Primary Health Care. We carried out in-person consultations with people closely involved in the Sustainable Development Goals task groups and those specifically involved with designing Aardram and the Family Health Centre concept in Kerala. We also carefully documented monitoring processes and existing data sources for monitoring in Kerala through fieldwork in multiple districts and by requesting inputs from various experts. Based on these inputs, trying to link global frameworks to local priorities and processes, we arrived at a shortlist of indicators. These indicators were ranked and discussed in a one-day meeting involving people from the state involved in grassroots implementation (in August, 2018).

Overall Considerations while doing the ranking

- This activity is guided by the Kerala 2020 SDGs – and our aim is to assess progress to this end, with no one left behind. Please use this as your overall frame.
- We need no more than about 30 overall indicators which can be used for monitoring, at the FHC level (population of ~30,000) but perhaps also at other levels as well.
- **OVERALL,** are we covering all the priority measurement areas of PHC/FHC services in Kerala?
    - ie. Are the current domains adequate? Do we need additional domains specific to Kerala (like LSGI inputs, community participation, technical efficiency)?
- **IN EACH DOMAIN,** are the selected indicators adequate to monitor/evaluate progress at the given level of analysis (Eg. Are the NCD indicators adequate to assess progress in rollout of services over time?
    - Do any indicators need to be considered together in order to reach the correct interpretation? If yes, is it better to exclude or include them?
    - Are there particular sub-populations (sentinel groups) that we should pay special attention to (tribal groups. Fisherfolk, the elderly)? If yes, do please note that.

Again, kindly refer the long list of indicators section and suggest replacements from the inventory for domains or suggest new indicators, whichever is preferable.

Domain specific considerations while doing the ranking – Non Communicable Diseases

We suggest that you think about the following questions when assessing indicators related to communicable diseases. This is just illustrative, please feel free to bring in your own concerns and reflections.

- Should the framework eventually include data on suicide rates?
- Should there be an indicator of physical activity?
- Should there be an indicator to look at salt intake, fruit and vegetable consumption etc?
- Do we need to consider a process indicator to look at activities targeted at health promotion, Adherence to COTPA, Health education classes etc?

Additional Criteria for ranking

Please consider the following priority matrix[1] to rank the indicators.

An indicator that is ranked 1 should have a response of YES to most of the questions to consider.
An indicator that is ranked 5 will have mostly NO responses.

Criterion	Definitions	Questions to consider
Sensitivity	System responses to changes will lead to noticeable changes in the indicator that can be accurately interpreted	• Does this indicator reveal whether the objectives of the system are being met?<br>• How would you interpret a change (increase or decrease) in the indicator measure?<br>• How much will the value of the indicator change in a short period of time?
Frequency	Changes in the system will be quickly reflected by changes in the indicator.	• Are changes in the system quickly reflected by changes in the indicator, or is there a significant time lag<br>• Is the frequency of monitoring correctly placed
Specificity	Indicator will accurately reflect changes in the system that it is intended to measure.	• Will any factors affect the indicator that do not reflect relevant changes in the system?
Feasibility	The data for the indicator are feasible to collect over time.	• How difficult/expensive is it to collect the data needed for the indicator?<br>• Are the required data routinely collected?<br>• How reliable are the data?
Purity	The data for the indicator cannot be manipulated, corrupted, gamed, or adjusted.	• How easy or difficult is it to manipulate or adjust the existing data?
Usability	The results of the indicator can (and will) be used to inform decisions.	• How closely does the indicator answer the measurement question?<br>• How easy or difficult is it to translate the results of the indicator into a decision?
Acceptability	Indicator is acceptable to key stakeholders and does not create political risks or concerns.	• Will all stakeholders agree on this indicator?<br>• Will policymakers understand what the indicator conveys?<br>• Are there any political risks associated with reporting this indicator?

[1] Adapted from: Measuring the Performance of Primary Health Care: A Toolkit for Translating Data into Improvement. Joint Learning Network for Universal Health Coverage, 2018.Available at: *http://www.jointlearningnetwork.org/uploads/files/resources/JLN_PHC_Measurement_Toolkit.pdf*

The George Institute for Global Health India

Ranking Sheet (*Please click on the check box once to select your response. If you click again, you can erase your selection*)
Please refer to the excel " Indicator short list" sheet for more information on these indictors and the "Indicator Long list "sheet for additional indicators

	Recommended Indicators	Ranking in terms of priority for Kerala	Justification for ranking
A	**Reproductive, maternal, newborn and child health**	1: high priority<br>5: low priority<br>1 2 3 4 5	
1	Antenatal care, four or more visits (ANC4) (%)	☐ ☐ ☐ ☐ ☐	
2	Full immunization coverage rate (%)	☐ ☐ ☐ ☐ ☐	
3	Malnutrition treatment coverage rate among children under five	☐ ☐ ☐ ☐ ☐	
4	Incidence of low birth weight among newborns	☐ ☐ ☐ ☐ ☐	
	Add indicator here		
B	**Communicable diseases**	1: high priority<br>5: low priority<br>1 2 3 4 5	
1	Blood smears examined as a proportion of population (%)	☐ ☐ ☐ ☐ ☐	
2	Proportion of households with positive vector-surveillance containers (%)	☐ ☐ ☐ ☐ ☐	
3	Prevalence of Acute Diarrhoeal Disease (ADD) among children under five (%)	☐ ☐ ☐ ☐ ☐	
4	Proportion of population using safely managed drinking-water services (%)	☐ ☐ ☐ ☐ ☐	
5	Tuberculosis (TB) effective treatment coverage (%)	☐ ☐ ☐ ☐ ☐	
6	Case detection rate for all forms of TB	☐ ☐ ☐ ☐ ☐	
	Add indicator here		
C	**Non Communicable diseases and Palliative care**	1: high priority<br>5: low priority<br>1 2 3 4 5	
1	Proportion of adults aged ≥15 years not smoking tobacco in last 30 days (%)	☐ ☐ ☐ ☐ ☐	
2	Prevalence of normal blood pressure, regardless of treatment status (%)	☐ ☐ ☐ ☐ ☐	
3	Prevalence of current alcohol use among persons aged 18+ years (%)	☐ ☐ ☐ ☐ ☐	
4	Prevalence of raised blood glucose among adults (%)	☐ ☐ ☐ ☐ ☐	
5	Cervical cancer screening rate	☐ ☐ ☐ ☐ ☐	
6	Number of patients presenting in health facility with mental illnesses	☐ ☐ ☐ ☐ ☐	

	Recommended Indicators	Ranking in terms of priority for Kerala	Justification for ranking
7	Proportion of patients receiving palliative care services (%)	☐ ☐ ☐ ☐ ☐	
8	Proportion of patients receiving one home care visit per week	☐ ☐ ☐ ☐ ☐	
	Add indicator here		
D	**Service capacity & Access**	1: high priority<br>5: low priority<br>1 2 3 4 5	
1	Proportion of patients referred from Family Health Centre (FHC) to higher levels	☐ ☐ ☐ ☐ ☐	
2	Proportion of Junior Public Health Nurse positions vacant at sub-centers (SCs)	☐ ☐ ☐ ☐ ☐	
3	Proportion of Staff Nurse positions vacant at FHC	☐ ☐ ☐ ☐ ☐	
4	Proportion of Medical Officer (MO) positions vacant at FHC	☐ ☐ ☐ ☐ ☐	
5	Proportion of facilities providing extended service hours	☐ ☐ ☐ ☐ ☐	
6	Proportion of patients receiving precheck service by Staff Nurse	☐ ☐ ☐ ☐ ☐	
7	Proportion of health facilities with Kerala-recommended core list of essential medicines available	☐ ☐ ☐ ☐ ☐	
	Add indicator here		
E	**Financial Risk Protection**	1: high priority<br>5: low priority<br>1 2 3 4 5	
1	Headcount ratio of catastrophic health expenditure	☐ ☐ ☐ ☐ ☐	
2	Proportion covered under a pre-paid scheme for financial protection	☐ ☐ ☐ ☐ ☐	
3	Out of pocket expenditures as Proportion of total current health expenditure	☐ ☐ ☐ ☐ ☐	
	Add indicator here		
F	**Governance & Stewardship**	1: high priority<br>5: low priority<br>1 2 3 4 5	
1	Proportion of LSGI funds spent on health and health related projects	☐ ☐ ☐ ☐ ☐	
2	Proportion of facilities that submit reports within the required deadline.	☐ ☐ ☐ ☐ ☐	
3	Proportion of Reporting Units (RUs) reporting in stipulated time period against total RUs, for P and L forms during a specific year	☐ ☐ ☐ ☐ ☐	
	Add indicator here		

A. What, if any, are indicators you feel absolutely should **<u>not</u>** be in Kerala's Aardram monitoring system?

B. What, if any, are indicators you feel **<u>absolutely should be</u>** in Kerala's Aardram monitoring system and are not here in the list?

C. Any other comments or considerations you'd like to submit?

Name :
Designation :
