# Supplementary material for: Monitoring Universal Health Coverage reforms in primary health care facilities: Creating a framework, selecting and field-testing indicators in Kerala, India
Source: PLoS One. 2020 Aug 3;15(8):e0236169. doi: 10.1371/journal.pone.0236169 (PMC7398520; doi:10.1371/journal.pone.0236169)
Supplement: Attachment — Submitted filename: Response to Reviewers.docx [file pone.0236169.s003.docx]

*We thank Reviewer 1 for the comments and support.*

**Reviewer 2** comments and author responses

1. **It would have been useful if authors present descriptive statistics (age, gender, occupation/specialisation and years of experience in the particular service) of the participants (or expert panel).**

We have added a table (see page 14, line 211) and text (see page 13, lines 201-208, underlined in the manuscript) that describe the characteristics of participants. The table and text are also reproduced below

**Table 2. Basic profile of experts participated in two rounds of modified Delphi method to develop monitoring indicators for primary care in Kerala.**

|  | Number (percentage) of participants) (N=25) |
| --- | --- |
| Age | |
| 30-40 yr | 5 (20) |
| 40-50 yr | 8(32) |
| 50-60yr | 7(28) |
| 60 above | 5(20) |
| Gender | |
| Male | 16(64) |
| Female | 9(36) |
| Domain of Primary Expertise | |
| Non communicable diseases, Injury and Palliative care | 8(32) |
| RMNCHA | 2(8) |
| Communicable disease | 4(16) |
| Health financing | 2(8) |
| Service capacity and delivery | 11(44) |
| Years of domain experience | |
| 5-10 yr | 6(24) |
| 10-20 yr | 10(40) |
| 20 years above | 9(36) |
| Job profile | |
| Field health worker | 3(12) |
| Primary care doctor | 2(8) |
| Program officer/Program consultant | 10(40) |
| Academicians | 4(16) |
| Senior state health administrator | 4(16) |
| District health administrator | 2(8) |
| Institutional Affiliation | |
| Department of Health services | 12(48) |
| National Health Mission | 5(20) |
| Department of Medical Education | 2(8) |
| Other (includes academic institutions, private facilities, multilateral institutions and state agencies) | 6(24) |

*Nearly half of the experts (48%) who participated in two rounds of the exercise were above fifty years of age and almost two-thirds were male (64%) (see Table 2). Most experts had a domain experience of more than ten years (76%) and nearly half of them (48%) were currently serving as state level specialist consultants or program officers (48%) for public health programs. Participants also included those working in a grassroots implementation like field-level health workers, and primary care doctors – almost all experts had experience delivering primary health care at some stage of their careers*.

2. **How many people were contacted and what is the rate of participation? At one place authors stated that a large group of experts were contacted but did not mention how many and what are their basic profiles. How did they reduce biased opinions? What is the selection procedure**?

This text has been added to the manuscript on pages 13 and line number 199-200. It is reproduced below

*A total of 31 participants were invited to participate in two Delphi rounds, out of whom 25 responded (a response rate of 80%, see Figure 2).*

The measures to reduce bias in opinion have been added in the manuscript on page 8 line number 169-174 and are reproduced below:

*To reduce bias in ranking, the tool consisted of specific information on consideration of how to rank an indicator* (*S1_File).* *Experts were encouraged to discuss with their colleagues in the respective domain about the relevance of individual indicators in a comprehensive monitoring framework. A team member visited the experts individually after sending them ranking tool to clarify and doubts in ranking procedure and reiterated the principles to rank them*

Information on sampling and the selection procedure has been added on page 7 line number 143-145, It is reproduced below. Profile information, as indicated above is given in table 2 and included in the manuscript on page 14.

*Eligibility criteria for participation were: experience of more than five years in relevant domain; first hand experience with primary care in Kerala; and past or present formal role in design and/or delivery of the FHC program*

*.*

3. **What kinds of scales are used to assess the agreement on the subjects/items/indicators? It is good if authors present some descriptive statistics of those results**

Details regarding the scale used are included in the manuscript on page 9 line number 175-181 and added to table 1 on page 10. The text is reproduced below

*Following standard convention and the procedure undertaken in prior ranking exercises [22], mean and median priority scores were calculated for each indicator by creating decision rules based on the distribution of ranks. For the final indicator list, indicators that received a median rank of 1 were included Table 1). Further, Indicators that received a median rank greater than 1 were included only if the mean rank of the indicator was higher than 2.5.*

4. **It will also benefit the readers to know at what level consensus or item consolidation was achieved in both the rounds. At what confidence level, authors have decided to include or exclude an indicator/item***.*

As is the convention, the level of consensus was determined quantitatively by obtaining mean and median of ranking of each indicator provided by the experts. We have decided to include or exclude an indicator/item based on the decision rule indicated on page 9, reproduced above.
